# Case Report: Non-microscopic surgical management of incomplete penile amputation

**DOI:** 10.12688/f1000research.23775.2

**Published:** 2020-09-22

**Authors:** Donny Eka Putra, Theddyon Bhenlie Apry Kusbin, Paksi Satyagraha, Stephanie Taneysa Widodo

**Affiliations:** 1 Urology Department, dr. Dradjat Prawiranegara Hospital, Serang, Indonesia; 2 Bethsaida Hospital, Tangerang, Indonesia; 3 Urology Department, Faculty of Medicine, University Brawijaya/Saiful Anwar Hospital, Malang, East Java, Indonesia; 4 Universitas Pelita Harapan, Tangerang, Indonesia

**Keywords:** traumatic, penile amputation, replantation, case report

## Abstract

**Background:** Penile amputation is an emergency urologic condition requiring immediate attention in order to maximize functional outcomes. Unfortunately, there is limited experience and publication of case reports describing the successful replantation of penis after incomplete amputation, especially in facilities without adequate microsurgical tools and means. We hereby present a case of penile amputation caused by a mechanical grass cutter and a discussion of its surgical management.

**Case description:** A 33-year-old Indonesian male presented to the emergency department with incomplete penile amputation six hours post injury. The patient has no prior medical history and presented with penile amputation due to a mechanical grass cutter trauma. He underwent immediate non-microsurgery reconstructive replantation of the penis, reattaching all visible vascular, corporal, and fascia layers. After replantation, the patient recovered well and showed preserved normal appearance and sensitivity of the penis. Subsequent Doppler ultrasound investigation revealed adequate arterial flow at the distal end of the anastomosis. The patient was discharged five days after surgery.

**Conclusion: **In the absence of microsurgical tools and means, the use of non-microsurgical replantation with an at least 2.5x loupe magnification should be the choice of treatment in the case of incomplete penile amputation. The technique showed good outcomes involving adequate functional and cosmetic restoration.

## Introduction

Penile amputation is an infrequent emergency in the field of urology that needs to be addressed immediately in order to maximize functional outcomes. Frequently, the injury is caused by self-mutilation during an acute psychotic episode. Other etiologies include secondary circumcision, violence, criminal assault, and accidental trauma ^1^. The management of such injury has shifted from the previous inevitable penectomy to a simple reattachment of the organ with re-implantation by microvascular techniques ^2^. In 1929, Ehrich *et al.* reported the first penile replantation using a macrosurgical technique. In 1977, Cohen *et al.* and Tamai *et al.* reported the first successful penile replantation by microsurgical techniques, which includes the re-anastomoses of blood vessels and nerves ^3, 4^. However, there is currently no universally accepted regiment to the repair of penile amputation ^5^. There is limited experience and publication of case reports describing the favorable outcomes of penile replantation, especially after incomplete amputation. This case report evaluates therapeutic approach as well as outcome of non-microsurgical replantation of incomplete penile amputation and reviews related literature to summarize the relevance of the current clinical experience ^6^.

## Case report

**Figure 1.**
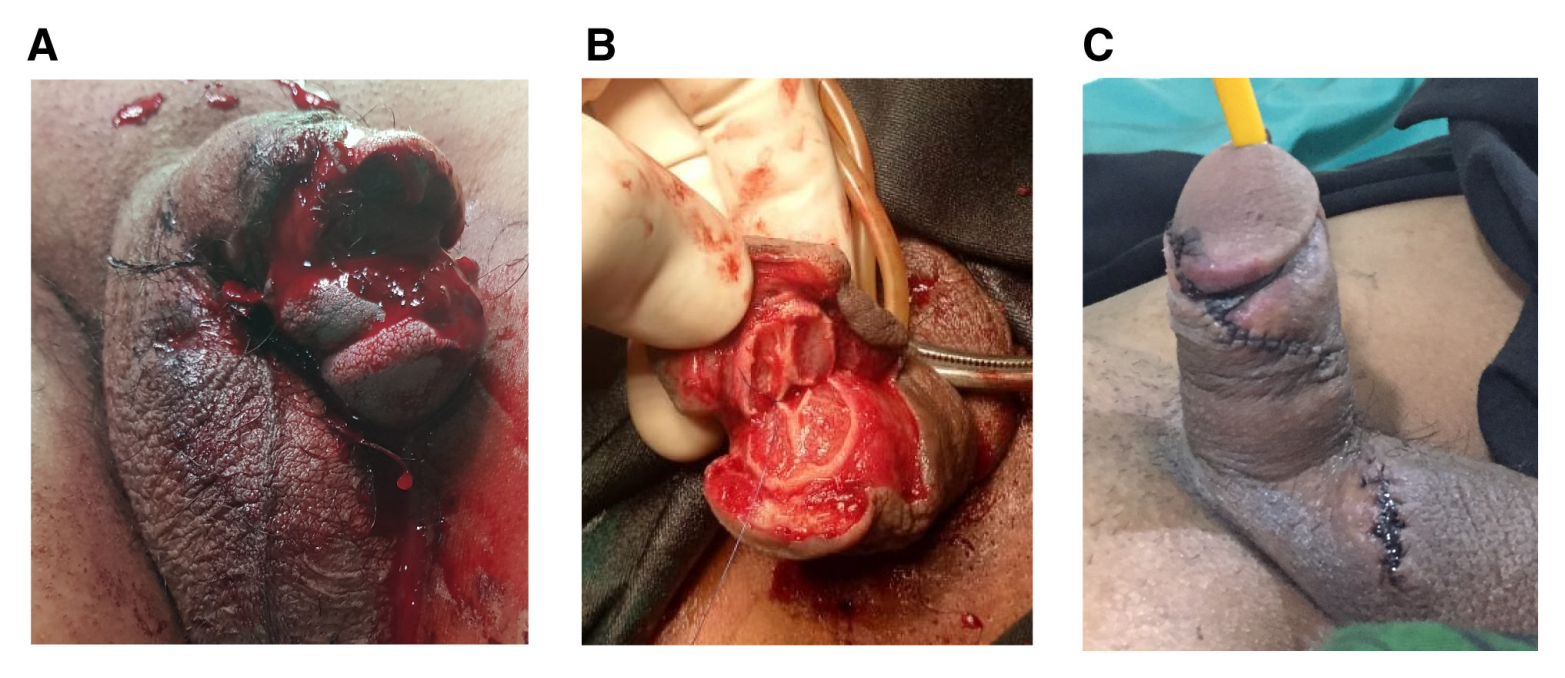
**A**: Penile amputation upon arrival to the emergency department. **B**: Wound exploration revealed cavernosal and spongiosal body rupture and allowed identification of deep penile arteries and superficial deep dorsal vein. **C**: Post-surgical evaluation at five days.

A 33-year-old married Indonesian male handyman with no significant past medical and psychiatric history presented to the emergency department of a type-C class rural hospital with partially amputated penis after sustaining a mechanical grass cutter injury six hours prior to the hospital visit. Pre-operation assessment in the emergency department was limited to penile amputation only, as we were unable to clearly define structural damages due to diffuse bleeding and excessive pain. At the time of patient arrival, trauma resuscitation protocol including airway, breathing, and circulation assessment was running. The patient was also given antibiotic anti-tetanus, blood specimen testing and on a 23G IV line to handle the earliest signs of shock if necessary. Although compression was immediately performed on the wound, bleeding still persisted ( Figure 1A).

At presentation, his vital signs showed blood pressure of 100/60 mmHg and heart rate of 100 bpm. Physical examination showed an amputated penis with diffuse bleeding. The detailed dorsal structures and urethra could not be evaluated properly. His scrotum and testicles were found to be intact. Laboratory tests were within normal values with a hemoglobin value of 14.5 g/dl.

Fluid infusion and antitetanic injection were administered and the patient was given emergency surgical management by the attending urologist. The patient was placed in the supine position and underwent general anesthesia with 200mg propofol, 50mg atracurium, 10mcg fentanyl, oxygen gas 1 liter/minute, and N2O 1 liter/minute. Saline irrigation bath was performed on the wound to allow visualization of all damaged structures. Surgical exploration revealed a complete detachment of cavernosal bodies, along with superficial and deep dorsal veins, dorsal and deep artery, and nerves injury. The spongiosal body sustained a partial rupture. The urethra was then found to be intact – no urethral mucosa was visualized – allowing insertion of a 16Fr foley catheter. The catheter was able to pass through without any resistance and therefore did not require any repair. Under loupe 2.5x magnification, the spongiosal and cavernosal bodies were sutured circumferentially at both ends with a vicryl 5-0 interrupted suture, followed by suturing the buck’s fascia with the skin with a vicryl 4-0 interrupted suture, followed by application of a pressure bandage. The deep arteries, dorsal arteries and nerve, and the superficial and deep dorsal veins were anastomosed with the available 2.5x loupe magnification. The surgery was completed within two hours and 15 minutes with total ischemia time of nine hours ( Figures 1B and 1C).

**Figure 2.**
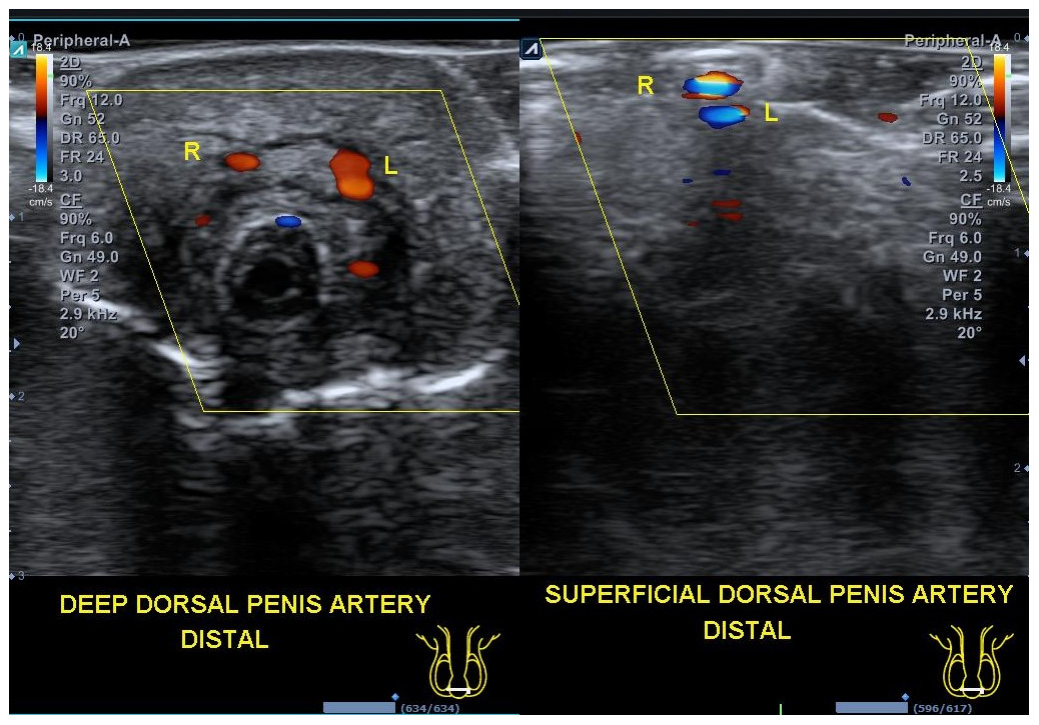
Post-surgical evaluation study at the fifth day with Doppler ultrasound showed adequate deep and superficial arterial flow at the distal end of the penis.

The patient was put on total bed rest until the third day post-surgery, with administration of intravenous 3 ^rd^ generation cephalosporine antibiotics (ceftriaxone 2gr/day), an analgesic (Ketorolac 25mg twice a day), and penile phototherapy (six hours a day) for five days. On the 3rd day after surgery we applied a new sterile dressing on the wound and observed no significant edema or pus or any leakage. On the fifth day after surgery, we adopted a post operative open wound treatment care plan. The catheter was also removed at the 5th day post surgery with close observation for hematuria or urinary retention. Five days after reconstruction, the penis showed no significant edema and swelling of the distal penile shaft, and sensation started to return gradually. The patient also underwent doppler ultrasound on day five to assess vascularization of penile distal to the injury. Evaluation with Doppler ultrasound showed adequate deep and superficial arterial flow at the distal end of the penis. On the fifth day post-surgery, the patient was discharged without urethral catheter following spontaneous micturition without difficulty in voiding ( Figure 2).

One month after reconstruction, the patient underwent further evaluation at a urologic clinic with good skin preservation and adequate wound healing. The patient was also evaluated with the International Prostate Symptom Score (IPSS) of 2, the International Index of Erectile Function (IIEF) score of 19, Quality of Life (QoL) score of 1, and Erection Hardness Score (EHS) grade II–III rigidity, along withfine penile sensation. There was no urinary fistula formation or difficulties in voiding. The patients felt satisfied with his condition.

## Discussion

Genitourinary injury accounts for as much as 33–66% of hospitalization of patients with external genital injuries ^7^. Males are more prone to genital injury than females due to anatomical differences. Male genitals are more exposed to violence, accidents, and extreme exercise. Of all genital trauma, 80% are caused by blunt injuries. Etiologies and classifications of genital trauma vary based on age (adult and pediatric), anatomical location, and the nature or mechanism of injury. Self-mutilation of the penis is one common etiology of adult genital injury and the majority of cases are associated with mental health problems ^7, 8^.

Penile amputation is an uncommon urologic emergency ^1^. It occurs due to a variety of etiologies. The majority of penile amputations are due to self-mutilation due to psychiatric disorders, which accounts for about 87% of all cases. Klingsor syndrome is a psychiatric disease involving self-mutilation characterized by paranoid schizophrenia along with command hallucination as well as disorders of eating such as anorexia and bulimia. The extent of penile injury ranges from minimal skin laceration to total amputation ^8^. A minority of reported cases arise from accidental industrial trauma, masturbatory trauma, and assault by spouses ^2, 9^.

The first documented case of penile replantation was reported by Ehrich in 1929. At that time, penile replantation was conducted on a traumatic injury using a non-microsurgical technique, which involves removal of all necrotic tissue, approximation of related structure, and introduction of a slip graft to cover the penis. A few days after replantation, there was reported hematoma at the glans. Two years later, the patient had urethral stricture and penile shortening with a normal-appearing penis ^10^. However, the organ was functional and apparently in normal shape with few scars. In 1977, Cohen successfully reported the first microsurgical penile replantation ^5^. A systematic review of literature between 1966 to 2007 revealed at least 30 successful penile replantations ^11^.

In 2017, Morrison *et al.* conducted a systematic review of 106 patients who underwent penile replantation. They proposed that penile replantation appeared to be safe and effective ^5^. Liu *et al.* (2019) reviewed 13 published case reports regarding penile amputation in the last five years. It showed that gross contamination or prolonged ischemia time are not factors in successful penile replantation, unless the injury sustained was severe ^6^. However, penile amputation still possesses a great challenge to surgeons due to the current lack of cases, standardized surgical techniques and post-surgical protocols ^6^.

Assessment of the final outcomes of penile replantation have varied widely and is often limited to subjective assessment of both surgeon and patient alike ^11^. This involves survival of the organ, good urinary stream, satisfactory cosmetic appearance and return of sensation as well as erection ^2, 5^. Many reports have defined the factors that contribute to favorable outcomes. To name a few, the duration of ischemia time, type and mechanism of injury, severity of injury, as well as microscope use at time of surgery ^5, 11^.

Many studies revealed that the ‘golden period’ within six hours post amputation is needed for satisfactory surgical outcomes, but Liu *et al.* (2019) reported adequate recovery of structure and functional capacity after microsurgical replantation with ischemia time exceeding 10 hours ^6^. In addition, microsurgical repair after 16 hours cold ischemia or injuries of greater than 24 hours has shown promising results ^12, 13^. The ischemia time of the patient treated in this report exceeded six hours (about nine hours), but the final outcome of the patient also showed adequate functionality and cosmetic restoration.

A review by Phonsombat *et al.* of 110 cases of penile amputation showed that gunshot injury (49%) was the most common cause, followed by stab injury or laceration (44%), and bite injury (7%). Surgical reconstruction after penetrating trauma of the penis might be technically easier due to better identification of related structures with intact margins. Blunt penile trauma, however, is more challenging due to the deformed anatomy and unmarked margins ^6, 14^. In our case, the injury sustained was due to a mechanical grass cutter at a factory.

Based on the severity of injury, penile amputation can be classified into complete and incomplete ^15^. There is no clear definition regarding incomplete amputation. Liu *et al.* showed that incomplete amputation with survival of vessels and nerves has a better prognosis compared to those with neurovascular damage ^6^. A retrospective analysis by Morrison *et al.* concluded that total amputation, increased amount of nerves conglutinated, and anastomosis of the superficial dorsal artery all bear significant association to positive outcomes. In their opinion, complete amputation of the penis tends to have better results because it enables the surgeon to access the neurovascular structurer more clearly. However, numeral illustration and clarification of vessels requiring anastomosis were not available from their data ^5^.

The preferred surgical techniques, either via microscopic or non-microscopic techniques, are still conflicting. Evaluation of two cases by Liu *et al.* showed that microsurgical repair was associated with better physical and psychosocial outcomes. Early anastomosis of penile neurovasculature is a critical factor that favors a successful outcome ^6^. Microsurgical techniques enable appropriate anastomosis or coaptation of structure, which allows better sensation and control of sexual function and leads to greater patient satisfaction. Jezior *et al.* reported that meticulous anastomosis of cavernosal arteries and dorsal structure was associated with erectile function ^16^. A contemporary report recognized the role of microsurgical revascularization in maintaining early and adequate penile blood flow in order to achieve the best appearance and erectile and voiding function outcomes ^11^.

Based on the characteristics of penile blood supply, it is possible to have a good outcome without the need for blood vessels to be re-anastomosed ^15^. Riyach *et al.* reported a case of incomplete penile replantation using non-microsurgical techniques. The deep penile arteries and superficial deep dorsal vein were not repaired. The outcome was good with a normal-appearing penis, good sensation, ability of penile erection, and ejaculation ^9^. They suggested that the spongiosal bodies may play a role in the arterial supply, venous drainage, and penile erection. Another successful non-microscopic penile replantation was reported by Mensah *et al.* The aforementioned case reported good voiding flow, cosmetics, and ability of penile erection. They stated that the corporal bodies might play a role in channeling penile blood flow. A review by Kochakarn (2000) concluded that both microsurgical and macrosurgical techniques constituted a good outcome after penile replantation. He reviewed 100 cases with ischemia time of up to 24 hours. The result was satisfying with adequate cosmetics and restoration of erectile ability. The most common complication was skin loss and urethrocutaneous fistula. He underlined that if there was not any microsurgical skills or facilities, penile replantation should be done macrosurgically, because it is proven to show good outcomes ^17^.

Moreover, Li *et al.* ‘s study involving 109 cases of penile replantation, 51% of which involved non-microsurgical repair, concluded that erectile dysfunction and urethral stricture were more common among patients who underwent non-microsurgical repair. Another report by Mendez *et al.* showed that a non-microsurgical approach led to necrotic skin, penile sensation loss, urethral stircture, and urethrocutaneous fistulas ^18^.

In our case, we carried out a non-microsurgical penile replantation in a patient with dorsal structure and cavernosal bodies rupture. As with the case report by Riyach *et al.*, the spongiosal bodies of our patient was partially spared. We used 2.5x loupe magnification to approximate the penile structure. On the fifth day post reconstruction, the penis showed no significant swelling of the distal penile shaft, and sensation started to return gradually. Our case demonstrated a good post-operative result with venous drainage restoration without a microsurgical technique. This raises questions about the role of the spongiosal body in penile blood flow and erection.

The ischemic time presented in our case was within six hours post injury, and we finished reconstruction in about two hours. Although we did not use a microscope to anastomose a large number of vessels, this led to a shorter ischemic time and therefore better overall outcomes. Moreover, distal penile injuries bear a greater challenge for vascular anastomosis in regards to the involvement of smaller vessels ^19^.

The limitations of this study include the unavailability of long-term outcomes reported in this study. After coming in for the one month follow-up, the patient ceased to show up for further monitoring. Other limitations include the lack of standardized and validated methods used to report study outcomes. There are currently limited clinical data depicting long term outcomes and functionality of heterogenous surgical repair methods. The strength of this study includes the successful management of a rare case of penile amputation with a partial sparred spongiosal body. Moreover, most cases of penile amputation comprise of complete amputation. ****

Penile amputation is an infrequent urologic emergency resulting from a variety of factors. This case report outlines the treatment of distal penile amputation with partial spongiosal injury. Although the gold standard treatment for said injury is a microscopic neurovascular reconstruction technique, non-microsurgical penile replantation with an at least 2.5x loupe magnification in a resource-deficient setting seems to yield adequate results.

## Data availability

All data underlying the results are available as part of the article and no additional source data are required.

## Consent

Written informed consent for publication of clinical details and clinical images was obtained from the patient.
