# Supplementary material for: Case Report: Non-microscopic surgical management of incomplete penile amputation
Source: F1000Res. 2020 Sep 22;9:681. Originally published 2020 Jul 7. [Version 2] doi: 10.12688/f1000research.23775.2 (PMC7542248; doi:10.12688/f1000research.23775.2)
Supplement: Peer review history

# Peer review: Reviewer response for version 2

**DOI:** 10.5256/f1000research.29017.r71791

**Published:** 2020-10-07

**Authors:** David J. Ralph

**Affiliations:** 1 Andrology Department, University College London Hospital, London, UK

I am happy now with the corrections.

Are enough details provided of any physical examination and diagnostic tests, treatment given and outcomes?

No

Is the case presented with sufficient detail to be useful for other practitioners?

No

Is sufficient discussion included of the importance of the findings and their relevance to future understanding of disease processes, diagnosis or treatment?

No

Is the background of the case’s history and progression described in sufficient detail?

No

Reviewer Expertise:

NA

I confirm that I have read this submission and believe that I have an appropriate level of expertise to confirm that it is of an acceptable scientific standard.

# Peer review: Reviewer response for version 1

**DOI:** 10.5256/f1000research.26235.r69356

**Published:** 2020-08-18

**Authors:** David J. Ralph

**Affiliations:** 1 Andrology Department, University College London Hospital, London, UK

- Need more anatomical description.
- Was this a division through the glans and distal corpora - urethral vessels were, therefore, supplying the glans?
- What about the dorsal neuromuscular bundle - please comment?
- Use the correct anatomy for each vessel, nerve, vein etc.
- Need a more subjective assessment of sensation and erectile function.

Are enough details provided of any physical examination and diagnostic tests, treatment given and outcomes?

No

Is the case presented with sufficient detail to be useful for other practitioners?

No

Is sufficient discussion included of the importance of the findings and their relevance to future understanding of disease processes, diagnosis or treatment?

No

Is the background of the case’s history and progression described in sufficient detail?

No

Reviewer Expertise:

Andrology and penile reconstruction

I confirm that I have read this submission and believe that I have an appropriate level of expertise to confirm that it is of an acceptable scientific standard, however I have significant reservations, as outlined above.

# Peer review: Reviewer response for version 1

**DOI:** 10.5256/f1000research.26235.r66594

**Published:** 2020-07-16

**Authors:** Omer A. Raheem

**Affiliations:** 1 Department of Urology, Tulane University, New Orleans, LA, USA

1. This is an interesting paper and adds to the current literature in penile injuries. I would recommend for publication; however, major revisions need to be added first.
2. Need to delineate and describe anatomical injury and orientation: ventral and dorsal? What is preop trauma/injury assessment and how it was managed at the emergency room level?
3. I understand microscopy was not available but it remains gold standard in penile injury repair and reconstruction especially when anatomizing penile vasculature and corporal tissues and urethral. However, surgical loops were used? Correct?  
4. How was urethral managed? Did you do preop UA, intra op cysto? Passing catheter? Any resistance? Did you repair urethra?
5. Describe in detail penile vascular injury? Was dorsal penile arteries, veins, and nerves all injures? Did you use intrap doppler to assess for arterial signals? 
6. Why it was not repaired? You also mentioned deep penile artery was not repaired? 
7. What is post op follow up? How many days? Months since the injury? Do you have SHIM or AUA SS assessed? Did he retain any erectile or ejaculatory function? Make clear in the paper?
8. Were other specialities like plastics present to assist or only urology?
9. What is the management of foley cath post op and wound care? 
10. Describe reconstruction sutures used and what fashion interrupted vs continuous? 
11. In your conclusion, you mentioned that microscopic repair remains gold standard; however, if there are limited resources, then non micro repair is possible. That’s ok. However you used surgical loops? Correct? 
12. Happy to write a short editorial to accompany this paper after revision is made.

Are enough details provided of any physical examination and diagnostic tests, treatment given and outcomes?

Yes

Is the case presented with sufficient detail to be useful for other practitioners?

Partly

Is sufficient discussion included of the importance of the findings and their relevance to future understanding of disease processes, diagnosis or treatment?

Partly

Is the background of the case’s history and progression described in sufficient detail?

Yes

Reviewer Expertise:

NA

I confirm that I have read this submission and believe that I have an appropriate level of expertise to confirm that it is of an acceptable scientific standard.

# Response

**Published:** 2020-07-28

**Authors:** Donny Eka Putra

**Affiliations:** RSUD dr Dradjat Prawiranegara, Indonesia

1. Thank you prof Raheem, we highly appreciate your feedback. We are deeply sorry for the delay in our respond. 

2. Need to delineate and describe anatomical injury and orientation: ventral and dorsal? What is preop trauma/injury assessment and how it was managed at the emergency room level?

The injury sustained resulted from a grass-cutter blade in the direction from the dorsal side of the penis. The injury consisted of dorsal artery nerves and veins, bilateral corpus cavernosum, and corpus spongiosum. The urethra was intact.

Pre-operation assessment in the emergency department was limited to penile amputation only, as we were unable to clearly define structural damages due to diffuse bleeding and excessive pain. At the time of patient arrival, trauma resuscitation protocol including airway, breathing, and circulation assessment was running. The patient was also given antibiotic anti-tetanus, blood specimen testing and on a 23G IV line to handle the earliest signs of shock if necessary.

The patient was then treated with compression bandage on the penis to minimize bleeding.

3. I understand microscopy was not available but it remains gold standard in penile injury repair and reconstruction especially when anatomizing penile vasculature and corporal tissues and urethral. However, surgical loops were used? Correct?  

Yes, we used surgical loupe with 2.5x magnification.

4.    How was urethral managed? Did you do preop UA, intra op cysto? Passing catheter? Any resistance? Did you repair urethra?

At the time of surgical exploration, urethra was found to be intact and the catheter was able to pass through without any resistance.  I did not do prep UA and i did not have the need to repair the urethra. 

5. Describe in detail penile vascular injury? Was dorsal penile arteries, veins, and nerves all injures? Did you use intrap doppler to assess for arterial signals? 

    Surgical exploration revealed dorsal penile veins and nerves injury along with corporal bodies. I did not use doppler US because it was not available during surgery.

6. Why it was not repaired? You also mentioned deep penile artery was not repaired? 

What I meant by ‘not repaired’ is that I did not repair it by micro-surgical means to create a proper end-to-end anastomosis. I however did an anastomoses by approximating the deep dorsal artery end with the available 2.5x loupe magnification.

7. What is post op follow up? How many days? Months since the injury? Do you have SHIM or AUA SS assessed? Did he retain any erectile or ejaculatory function? Make clear in the paper?

The patient was discharged from the hospital on the 5th day after surgery. At day 5, the patient underwent doppler ultrasound to assess vascularization of penile distal to the injury. The patient was discharged without catheter following no difficulty in voiding. At one month after surgery, the patient was evaluated with IPSS,  IIEF, and EHS score. 

Yes, the patient retained erection and ejaculation function at the level of grade 3 EHS, IIEF score 19, IPSS score 2, QOL 1.

8. Were other specialities like plastics present to assist or only urology?

No, Only Urology.

9. What is the management of foley cath post op and wound care? 

On the 3rd day after surgery we applied a new sterile dressing on the wound and observed no significant edema or pus or any leakage. On the fifth day after surgery, we adopted a post operative open wound treatment care plan. The catheter was also removed at the 5th day post surgery with close observation for hematuria or urinary retention.

 10.    Describe reconstruction sutures used and what fashion interrupted vs continuous? 

I used vicryl 5/0 interrupted suture on the corporal bodies and 4/0 vicryl interrupted suture on fascia and skin.

11. In your conclusion, you mentioned that microscopic repair remains gold standard; however, if there are limited resources, then non micro repair is possible. That’s ok. However you used surgical loops? Correct?  

Yes. My apologies, i will revise my conclusion with the recommendation of doing repair with at least a 2,5x surgical loupe like the way i treated the patient in the paper. 

12. Happy to write a short editorial to accompany this paper after revision is made.

Thank you, we highly appreciate the time you spent to review our paper. We would love to have your short editorial to accompany our paper.
